# Hair Dyes

**Published:** 1873-01

**Authors:** 


### HAIR DYES.

It would be a good and safe idea to pay no attention whatever to any newspaper statement in reference to health unless the source of the information were given, then the reader could exercise his own judgment as to the amount of credence to be given to any assertion made.

It is well-known that, in the larger cities, the making up of items for a newspaper is intrusted almost entirely to young men whose qualifications are measured by their literary ability, and who have no information beyond that which mere reading gives. These persons see a statement that is entirely against received opinions, and without any means of knowing the standing of the writer they forthwith put it in print, and it goes forth as of authority. Recently an itemizer in an evening daily, in reference to hair dyes, says, "There is not a particle of danger from that source"—meaning the absorption of poisonous matter through the skin—calling the contrary opinion, "unscientific twaddle;" this, too, in the face of a multitude of authenticated facts that persons using hair dyes have been afflicted with various forms of paralysis and convulsions, as will necessarily be the case when the dye contains one of the most poisonous substances—acetate of lead. Even drinking water from a leaden pitcher will poison a man; much more will it be done by rubbing the liquid form of the poison into the skin.

